# Health and economic benefits of energy, urban planning, and food interventions that lower greenhouse gas emissions

**DOI:** 10.1097/EE9.0000000000000404

**Published:** 2025-07-01

**Authors:** Mary B. Rice, George D. Thurston, Skye S. Flanigan, Vanessa B. Kerry, Lisa A. Robinson, Wuyue Yu, Ebba Malmqvist

**Affiliations:** a Center for Climate, Health and the Global Environment (C-CHANGE), Harvard T.H. Chan School of Public Health, Boston, Massachusetts; b Department of Medicine, New York University Grossman School of Medicine, New York, New York; c Center for Health Decision Science, Harvard T.H. Chan School of Public Health, Boston, Massachusetts; d Division for Occupational and Environmental Medicine, Lund University, Lund, Sweden

**Keywords:** Interventions, Public policy, Climate mitigation, Fossil fuel combustion, Diet, Benefit-cost analysis, Economic evaluation, Air quality, Air pollution

## Abstract

Public health can be immediately and substantially improved by policies that also mitigate climate change over the longer term. However, implementation of these policies has been slowed at least in part by doubts and lack of awareness of these health co-benefits. To address this barrier to progress, we demonstrate how an illustrative set of interventions led to environmental, health, and economic benefits, in addition to mitigating climate change. These case studies include the closure of a coal coking plant near Pittsburgh, PA, USA, which was followed by substantial immediate and longer-term reductions in respiratory and cardiovascular health conditions in the affected local community; the health and economic benefits associated with the Barcelona, Spain Superblock program and, the air quality, health, and economic benefits from air pollution initiatives implemented in China. While improvements in air pollution are among the most obvious examples of the co-benefits achievable through climate-friendly interventions, others that reduce greenhouse gas emissions, such as the sustainable food systems in Sweden, forest conservation in Tanzania, and a plant-based food program in New York City, further illustrate how such initiatives can align with better nutrition, economic gains, and improved health. We conclude that more assessments of such interventions are needed internationally to more widely document their health and climate benefits and thereby motivate greater implementation of these interventions. Now is the time to showcase how we can improve the public’s health and well-being, while also protecting our planet, the only home future generations will have.

## Introduction

While improving environmental conditions through air quality and climate mitigation actions will substantially enhance health and quality of life, progress has been slowed at least in part by doubts and lack of awareness of these benefits. However, as Francis Bacon famously said, “The best proof is experience.” Therefore, to support the 2nd Global Conference on Air Pollution and Health hosted by the World Health Organization and build on a prior ISEE-sponsored Workshop Report,^1^ we present real-world mitigation case studies where environmental and civic planning interventions led to documented health and economic benefits.

The creation of policy-relevant evidence that supports the translation of scientific evidence into societal action has always been a key goal of public health research. For example, pioneers in public health documented the need for improved urban sanitation and safer workplaces,^2,3^ and later provided the evidence for meaningful tobacco control.^4^ Today, researchers in public and environmental health similarly face battles for clean air, safe chemicals, and climate mitigation and adaptation to save lives and improve quality of life.^5^ For example, one systematic review of the available evidence of health co-benefits of climate mitigation indicates that achieving net-zero emissions across different sectors (i.e., transport and energy) would generate considerable health benefits in 98% of the studies considered, primarily through improved air quality.^6^ Delaying these actions represents a missed opportunity to improve human health.^7^ In this commentary, we provide several examples of such translational research evidence, with the hope that it will encourage further and more comprehensive documentation of science to policy climate mitigation activities, thereby contributing to the larger WHO goal “*Health in all Policies.”*^8^

## Description of the process

To inform this commentary, the authors searched online databases and relevant literature to identify illustrative examples of interventions with climate mitigation benefits that evaluated both the local health and economic benefits. They reviewed the Pathfinder Initiative Evidence Bank by sector and health effects to identify the three key categories of cases where local public health benefits were observed within the first few weeks to months of GHG emission intervention implementation: Transition to Cleaner Energy Sources (i.e., energy, electricity generation, and industry sectors with Air Quality Health Effects on Pathfinder), Cities and Urban Planning (i.e., transport, buildings, and cross-cutting sectors with Air Quality Health Effects on Pathfinder), and Food Systems and Agriculture (i.e., agriculture, forestry and other land-use sector with diet and/or livelihood benefits from Pathfinder). To estimate the city-level policy-, case-, or intervention-specific health and economic benefits, the authors selected city/local-level cases from the Pathfinder database and searched for literature examining city/local-level effects related to national/sub-national policies. The authors also included a literature search of English full-text articles that assessed air pollution interventions and health effects to identify examples not included in the Pathfinder database. Additionally, they engaged with a network of subject-matter experts specializing in assessing these interventions to solicit relevant resources and insights. The authors further reviewed and selected illustrative example cases where (1) a clearly defined intervention was implemented; (2) there is clear evidence of local public health improvement in the following time period; (3) there is a reduction in GHG emissions from the Pathfinder database, related literature, or calculations by the authors based on additional data; (4) monetary values for at least some impacts can be directly extracted or calculated based on reports and/or literature. The findings were summarized in the manuscript and accompanying tables, which document both the health outcomes assessed and economic values. The sources of these estimates, including supplemental calculations by the project team, are documented in the references and footnotes and available upon request to ensure methodological transparency and facilitate replication by other researchers.

## Real-world case studies

In this commentary, we selected 11 illustrative examples from seven different countries to illustrate how public health improvements can motivate climate actions. We reviewed the real-world case studies from the Pathfinder Initiative Climate & Health Evidence Bank, searched published literature on air quality interventions and health benefits, and engaged with a network of subject-matter experts to solicit relevant insights. We document the benefits by assessing the health impacts of these interventions. Economic and financial assessment of these interventions that evaluates and compares the overall costs, savings, and benefits is reported when possible to demonstrate overall cost-effectiveness and net benefits. While the effects of reducing greenhouse gas emissions are global and occur over the long term, these health benefits are local and observable within weeks to months after intervention implementation. Combined, such evidence of health and economic benefits can act as an effective lever for action by demonstrating the extent to which communities achieve net welfare improvements and enhanced revenues from these actions and may help motivate non-health actors to invest in health and well-being.

The adverse effect of air pollution exposure on health and its related societal costs is a strong example and opportunity to motivate intervention that can improve health outcomes and create near-term economic benefits, as well as mitigate climate-forcing pollution. Past interventions in energy and urban planning provide multiple examples of the health benefits of air quality improvements. They not only relate to traffic activity but also to industrial and power plant operations. For example, in Pittsburgh, USA, local residents had been subjected to high levels of exposure from a coal coking plant, as part of steel production. When the plant finally closed after years of protests, the cardiovascular health in the area immediately improved after the week of closure and continued improving over the following years. As summarized in Table 1, health scientists documented a subsequent reduction in emergency hospital visits, which included a drop in the number of cardiovascular and asthma cases within weeks from the closure in the surrounding communities that experienced the dramatic pollution decline, as well as longer-term health trend of steadily reduced cases in these communities. Similar health metric improvements were observed in areas where coal power plants were shuttered, as noted in Table 1 for Chicago, the US, and China. In addition to the reduced societal health care costs, these local health improvements were likely also accompanied by increased productivity and income for those living in the areas with improved air quality.^25,26^

**Table 1.** Illustrative examples of health benefits from climate interventions

Case studies	Timeline and challenges	Health impacts	Economic values
Transition to cleaner energy sources			
1. Local emission reduction due to the closure of steel industry coking plant used in Pittsburgh, PA—In 2016, the Shenango Coke Works plant was closed, eliminating an estimated 1.3 million tons/yr of CO_2_ emissions^9–11^ and improving air quality for the surrounding community.	The Shenango Coke Works was closed on January 6, 2016. Residents had been subjected to high levels of exposure and protested for years before the closure. The community pressure and the decline of domestic steel industry hastened the shutdown.^12^	Health improvements: Over the 3 years after the shutdown: ● 6,744 fewer cardiovascular emergency department visits. ● 724 fewer cardiovascular hospitalizations^13^ in the local community. Mortality avoided: not reported.	Health improvements: Over the 3 years after the shutdown: ● US$7,830,000 emergency room medical cost savings^14^ ● US$12,249,000 hospitalization medical cost savings plus productivity gains^14^ in the local community. Mortality avoided: not reported.
2. Local emissions reductions due to the closure of three coal-fired power plants in Chicago, USA—In 2012, these closures resulted in health and economic benefits for neighboring communities, in particular for children under the age of 4.	Between March and August 2012, 3 coal-fired power plants in or near Chicago closed. Local community members had long been concerned about the pollution and health risks. The closing of these plants was achieved after over a decade of local community organized actions, with the confluence of Chicago’s rebranding efforts, the first contested mayoral election in over 20 years, and a changing global market and national regulatory environment making coal less competitive. ^15^	Health improvements: In the 5 years after the closure, on an annual basis: ● 406 fewer asthma emergency room visits (children under age 4) per year.^16^ ● 363 fewer school absence days per year.^17^ Mortality avoided: ● A study of retired US coal-fired plants (1999–2013) found 1.7% lower monthly mortality rate (age >65) and 3.6% lower age-adjusted mortality per closure relative to control counties.^18^	Health improvements: In the 5 years after the closure, on an annual basis: ● US$217,000 emergency room medical cost savings per year. ● US$431,000 parental productivity gains and improved learning per year.^19^ Mortality avoided**:** not reported.
3. Local emissions reductions due to the closure of 13 coal-fired power plants in China—Between 2000 and 2015, these power plants closures resulted in improved health outcomes for downwind communities.^20^	China has closed around 150 large coal plants since 2000, as well as numerous smaller plants that used outdated technologies and produced substandard emissions Concerns over air quality and availability of alternative energy motivated these policies. China faces economic and social challenges in coal transition, for example, economic stress, workforce development, and industrial reform in resource-dependent cities. Policies also have faced resistance from coal interests and experienced reversals.^21^	Health improvements: ● 7% reduction in sickness incidence in downwind vs upwind residents, 5–9 years after closure. ● Lower incidence of obesity and hypertension, 5–9 years after closure. ● Another study estimated each coal-fired plant in Beijing was associated with 279 bronchitis cases annually.^22^ Mortality avoided: ● In a Beijing study, the closure of each coal-fired plant was associated with 292 premature deaths averted annually.^22^	Health improvements: ● CN¥221,000 medical expenditure savings based on other studies, for 13 power plant closures.^20,22^^–40^ ● In Beijing study, the closure of each coal-fired plant is estimated to save US$7,100,000 in medical costs for bronchitis per year.^22^ Mortality avoided: ● In Beijing study, closure of each coal-fired plant is estimated to lead to benefits of $127,000,000 for reduced premature mortality per year

The estimates use reported data and have not been adjusted for comparability or inflation.

Case 1: The health improvement estimates were based on the following assumptions: years 2016–2018 and a study area of 75,000 near Pittsburgh; changes in hospitalization and emergency department visit rates as reported in the study and aggregated over the study period; US EPA regulatory valuation functions ($1,161 per ER visit case for cardiac outcomes in 2015 USD and $16,918 per hospital admission case for cardiac outcomes in 2015 USD) used for per case economic values.

Case 2: The health improvement estimates were based on the following assumptions: 48 zip codes were included for the study, with a mean population size of 57,743 and 6.8% in 0–4 years of age in 2009, and elementary schools in 15 school districts; intervention effect size per year as reported in the referenced studies; US EPA regulatory valuation functions ($534 per ER visit case for asthma in 2015 USD and $1,186 per day of school loss in 2015 USD from productivity of parent and lost learning) used for per-case economic values.

Case 3: The health improvement estimates were based on the following assumptions: In the case of 13 coal plant closures, a population size of 3,592, with a total number of 13,507 observations (unit of observation is person-year); the study reported an average probability of being sick in the past 4 weeks was 14.2% between 2000 and 2015 and was reduced by 7% (50% of its mean), or 945.5 cases. The valuation of estimated changes in morbidity focused on the reduced incidences of chronic bronchitis, which accounts for the largest economic benefits of avoided morbidity.^22^ The number of chronic bronchitis cases avoided was estimated by assuming that they account for a similar percentage in the incidence of sickness reduced from the shutdown of coal power plants as they do in national hospitalizations around the time of intervention (1.6% in 2008).^23^ The medical expenditure per person-year from literature (CN¥14,594 per chronic bronchitis person-year in 2010 CNY^24^) was used for per case economic valuation. Monetary benefits from the coal plant closures in Beijing were based on the estimated benefits of avoided premature deaths and chronic bronchitis in the coal-to-gas intervention in 2011 USD and derived from the proportional increase of cases avoided by the closure.^22^

Other clean air initiatives (see Table 2) such as environmental policies like China’s Blue Sky Defense Battle initiative, have also significantly cut pollution-related diseases and generated substantial economic benefits while reducing climate-forcing carbon dioxide emissions. Similarly, even short-term interventions have been shown to have health improvements, as seen in the City of Beijing, China ahead of their 2008 Olympics, when stricter environmental regulations immediately led to improved air quality during the Olympics that prevented premature cardiovascular and respiratory deaths.

**Table 2.** Examples of health benefits from climate interventions

Cities and urban planning			
Case studies	Timeline and challenges	Health impacts	Economic values
Barcelona superblocks—a concept for sustainable mobility developed by the city administration in 2016, with the first Superblock built in 2017. There are plans for 503 Superblocks, with plans also for a green corridor *Eixos Verdes*.^27^	The first superblock in Barcelona was implemented in the Born neighborhood in 1993, and in Gràcia neighborhood in 2003. In 2015, a city-wide plan was approved to create 500 superblocks by 2030. The project changed from square ideas to green corridors for better public acceptance and political discourse of urban design (car vs. people). Superblocks initially faced concerns from residents, businesses, and politicians, primarily about potential traffic disruption and the possible negative impacts on local businesses. Since implementation, public sentiment has shifted, due to reduced traffic and improved pedestrian mobility. A study found greater electoral outcomes for politicians supporting Superblocks in districts that had implemented Superblocks.^28^	Health improvements: ● 5% SDQ total difficulty scores and 6% reduction in hyperactivity/inattention scores.^29^ ● Perceived increased well-being by 55% of men and 45% of women.^27^ Mortality avoided: ● Approx 667 premature deaths annually for 503 Superblocks if implemented.^30^	Health improvements: not reported. Mortality avoided: ● Estimated annual benefits of €1.7 billion annually due to longer life expectancy if all 503 Superblocks are implemented.
London Low Emission Zones—In 2003, London implemented a low emission zone (LEZ) and Congestion Charging Scheme (CCS) to reduce congestion and air pollution in urban areas. A Low Emission Zone covers most of Greater London with the Ultra Low Emission Zone (ULEZ), was implemented in 2019. ULEZ has reduced road traffic and has reduced NO_2_ by 12.4% and PM_10_ by 27% since 2019.^31^	In 2003, London implemented a LEZ and CCS to reduce congestion and air pollution in urban areas. ULEZ, implemented in 2019, was expanded from 2021 onwards and now covers the most central parts of the City.^32^ The LEZ initially faced resistance, with only 39% of residents supporting it before its implementation in 2003. However, public support rose to 59% within months, due to reduced traffic and livability. By 2025, the ULEZ now cover the entire core of the City.^32^	Health improvements: ● ~1.1 million air pollution-related hospitalizations avoided by 2050.^33^ ● 8% reduction in asthma and bronchitis. ● 12 cases per 10,000 persons reduction in respiratory admissions.^34^ ● 6.5% reduction in anxiety. ● 18% reduction in sick leave.^31^ Mortality avoided: ● 183 years of life per 100,000 population. ● 1888 years of life were gained.^35^	Health improvements: ● ~UK£15 billion in savings by 2050 for reduced hospitalizations.^33^ ● UK£480 million in yearly savings for COPD. ● UK£15.5 million in yearly savings for sick leave.^34^ ● After accounting for costs and revenues, net benefit of ~ >UK£963 million in Greater London.^34^ Mortality avoided: not reported.
Milan Traffic Scheme—Introduced in 2008, this scheme restricted vehicles entering the city, resulting in a decrease in traffic accidents and economic benefits for the city.^36^	In January 2008, the municipality of Milan introduced a road charging scheme, and a public consultation in 2011 indicated strong (79%) approval for the congestion pricing program. In 2012, the fees and vehicle types were slightly amended. In 2022 and 2023, the scheme included restrictions on more polluting vehicle types and an increase in rates respectively.^37^	Health improvements: ● 18.8% decrease in traffic incidents inside the boundary.^38^ ● Changes in injuries not reported. Mortality avoided: not reported.	Health improvements: not reported. Mortality avoided: not reported. Other ● Increased gross revenues to €30 million in 2012 from €12 million in 2008.^39^
Blue Sky Defense Battle initiative—From 2018 to 2020, China implemented the 3-Year Action Plan for Winning the Blue Sky Defense Battle. This analysis addresses the Action Plan in Chengdu.	In 2018 the state released the 3-Year Action Plan, in 2019 they deepened their measures to include industrial upgrades, green transportation, and a focus on major polluters. In 2020, they focused their energy on the evaluation progress and monitoring systems. The program faced challenges such as seasonal temperature inversions, industrial dependency of the region, a surge of vehicle ownership, enforcement limitations, and the reluctance of public engagement and awareness.	Health improvements: In 2019-2020 ● 5,788 respiratory hospitalizations avoided ● 3,698 cardiovascular hospitalizations avoided. ● 94,223 Pediatric outpatient visits avoided. ● 233,723 internal medicine outpatient visits avoided. ● 13,550 chronic bronchitis avoided. Mortality avoided: ● 2,564 premature deaths.^40^	Health improvements: ● Health benefit is worth ¥9.79 billion.^40^ Mortality avoided: not reported.
2008 Beijing Olympics Air Pollution Reduction—Beijing took measures to clean up air pollution ahead of the games. The result was immediate air quality improvements (60% reduction in SO_2_, 48% reduction in CO, 43% reduction in NO_2,_^41^ and 30% reduction in PM_10_.)^42,43^	Multisectoral air quality regulations were implemented during the Olympics and subsequent Paralympics from July to September 2008 as a condition for hosting the 2008 Olympic Games.^41–43^ The substantial air quality improvement was temporary and air pollutants increased to reach or exceed preintervention levels during the post-Olympic period after the relaxation of the pollution control measures.^41^	Health improvements: ● Improved respiratory function^44^ and reduced inflammation^45^ during Olympics. Mortality avoided: ● Prevented 196,000 cardiovascular and respiratory deaths. ● Decrease of 10% in PM_10_ was associated with a 7% decrease in deaths per 100,000 people.^42,43^	Health improvements: not reported Mortality avoided: ● Prevention of premature deaths results in estimated economic benefits between US$58 billion and US$1 trillion per year.^42,43^

The estimates use reported data and have not been adjusted for comparability or inflation.

**Figure 1.**
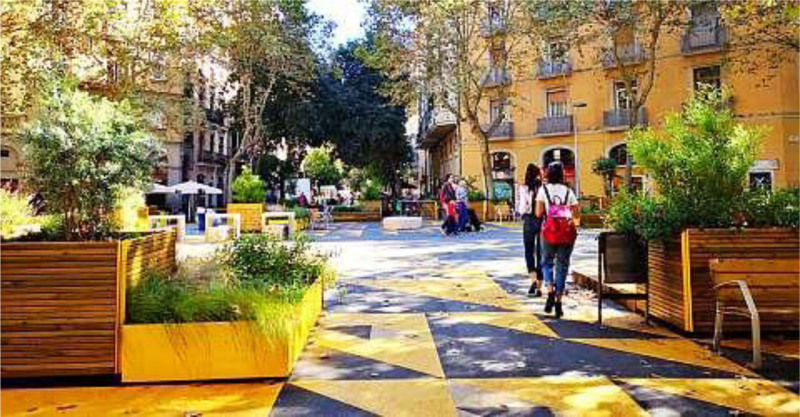
Example of Barcelona Super Block. Adapted with permission from Barcelona.de^48^

During the last decade, there have been multiple examples^46,47^ of healthy urban planning interventions that also reduced climate-forcing pollutant emissions, such as carbon dioxide, including initiatives in Barcelona, Milan, and London (see Table 2). Barcelona introduced superblocks where some streets experienced greatly reduced motorized traffic while increasing active travel instead. Those restrictions were complemented with efforts to make the streets more livable, such as adding plants and seating (Figure 1). This effort was made to improve air quality, green spaces, heat, noise, and physical activity. The intervention was demonstrated to improve self-reported well-being,^27^ and researchers estimate that a fully implemented superblocks program would avert 667 deaths per year in Barcelona, with a value of 1.7 billion EUR.^30^ London has taken another approach by creating zones where polluting vehicles are restricted from the city’s Ultra Low Emission Zones, which has not only reduced air pollution levels throughout the city^49,32^ but has had the additional co-benefits of decreasing adverse health effects^50^ and increasing children’s active travel to school.^51^ Documenting the economic, quality of life, and health benefits helps counterbalance the economic arguments often made against incorporating such health- and climate-friendly interventions.

While improvements in air pollution are potentially among the best-documented examples of the co-benefits achievable through climate-friendly interventions, examples across more sectors are emerging. Other interventions that reduce gre enhouse gas emissions, such as the sustainable food systems in Sweden, forest conservation in Tanzania, and a plant-based food program in New York City, further illustrate how such initiatives can align with better nutrition, economic gains, and improved healthcare access (see Table 3). While these cases demonstrate economic improvements, there is as yet little documentation in the literature of the full scope of health benefits from food system and agriculture interventions that logically improve overall health. To help aggregate the examples across disciplines, spur action for climate, health and co-benefits, and outline areas for further study, we outline a series of illustrative cases. Many of these case studies showcase how environmental and climate interventions can drive economic benefits, reduce health burdens, and enhance the overall quality of life.

**Table 3.** Illustrative examples of health benefits from climate interventions

Food systems & agriculture			
Case studies	Timeline and challenges	Health impacts	Economic values
Sustainable school meals in Sweden—In 2019, a small intervention was conducted for 4 weeks. Switching to sustainable menus reduces GHG emissions by 40% without negative effects on food waste, consumption, or cost.^52^	Sweden has a long history of prioritizing sustainability in public food procurement and in 2019, this 4-week intervention prioritized having lower dairy and meat consumption, higher plant-based proteins, and more locally sourced ingredients. The challenges faced were related to the cultural resistance as the traditional Swedish diet includes a lot of meats, training of the kitchen staff who did not have a lot of experience with plant-based cooking, ingredient constraints as some were more expensive than buying bulk, and a lack of evaluation framework for this novel intervention.	Health improvements: ● Met the same or better nutritional standards for school lunch programs. Mortality avoided: not reported.	Health improvements: not reported. Mortality avoided: not reported. Other: 11% cost reduction.^52^
Ntakata Mountains Forest Protection Programme—Established in 2016, this program aimed to create integrated solutions to create healthier fisheries, families, and forests and trained 20,000+ farmers in Climate-smart Agriculture. It resulted in hundreds of thousands of hectares of woodlands being protected, improved water quality at Lake Tanganyika, increased employment and revenues allocated to community health programs and educational initiatives.^53^	In 2016 & 2017, the program was established, and planning focused on community engagement. In 2018 & 2019 they implemented new frameworks for carbon credits and focused on climate-smart agricultural training. In 2020 & 2021, they focused on scaling up and diverssifying the initiative, and from 2022 to present they have been focused on impact assessments. The challenges include land-use conflicts, the economic pressures of balancing short-term needs with long-term conservation, capacity building with community members, and monitoring and verification of the impacts of the program.	Health improvements: ● Covered the medical expenses of ~26,400 people. ● Established two pharmacies, two ward hospitals, and four village dispensaries. ● Reduced maternal & neonatal deaths.^53^	Health improvements: not reported Mortality avoided: not reported. Other: ● Generated US$1,570,000 through carbon credits. ● 50% increase in crop yields.^53^ ● US$5,746,117 earned by communities in 2023.^54^ ● In 2022, households increased in 7 out of 11 staple assets from 2016. ● In 2022, 75% of households stored crops postharvest (vs 64% in 2016). ● 66% of farming households reported low postharvest losses (vs 54% in 2016).^55^
NYC Plant-Based Meal Program—In March 2022, this hospital initiative was introduced to encourage better eating that is less harmful to the planet. As of March 2024, over 1.2 million meals have been served, all while creating health benefits for patients, continuing patient satisfaction, lowering carbon emissions by 36%, and reducing costs for the hospital system.	In 2022 this program launched across NYC hospitals serving plant-based meals to patients. By 2024, the program had served over 1.2 million plant-based meals with a high satisfaction rate, cost savings, and reduced carbon emissions. The program is still ongoing. The challenges include maintaining nutritional standards, logistical challenges of scaling up the distribution, and cultural acceptance given the diverse patient population preferences.	Health improvements: ● Approval rating of 90% among patients Mortality avoided: not reported.	Health improvements: not reported Mortality avoided: not reported. Other: ● Cost savings of 59 cents per meal in 2023.^56^

The estimates use reported data and have not been adjusted for comparability or inflation.

## Conclusions

Public health measures that limit increases in global temperature and preserve nature can lead to substantial improvements in health and longevity that persist far into the future. Such mitigation and adaptation efforts also lead to significant immediate gains when health co-benefits are considered, as exemplified by the estimates provided in the tables. Reducing hazardous emissions prevents morbidity and premature deaths, which in turn saves health care costs and boosts productivity in addition to increasing overall welfare. Perhaps politically more important, research suggests that the less tangible effects of reducing these risks are highly valued by society. There are numerous interventions beyond the few outlined in our tables, but because the health impact on the community “with” versus “without” the intervention has not been comprehensively assessed for most interventions, we have not been able to enumerate them. The cases highlighted in this commentary and detailed in Table 1, all illustrate health and economic benefits that resulted from the initiatives. Many of these case studies underestimate the value of the health benefits because they did not consider the value that individual members of the public place on averting these health effects, including avoiding associated pain and suffering. Indeed, reducing asthma incidence may be valued by people at 10 times more than the averted medical costs and productivity losses alone.^57^ The value individuals place on reducing their own mortality risks often exceeds the value of averted costs by orders of magnitude and deserves more attention in these studies.^25,58,59^

At this critical time for climate mitigation and adaptation, it is essential to communicate the near-term benefits of action. There are diverse and substantial health, economic, and quality of life benefits of local public health actions that reduce emissions.^1^ Further replication of such public policy interventions as we present here can lead to improved health and economic outcomes that will, in turn, help us learn from the success of others, inspiring still more such actions. Researchers, policymakers, businesses, civic planners, and communities must partner together to demonstrate the value of such climate-friendly public policy actions. More economic evaluations of interventions are clearly needed, that employ best practice methods to document the health and climate benefits, including implementation costs in relation to immediate and long-term valuations of health benefits.^60–62^ Now is the time to showcase how and why we cannot wait and to enable government and policymakers to improve the lives of their constituents, while also protecting our planet, the only home future generations will have.

## Conflicts of interest statement

The authors declare that they have no conflicts of interest with regard to the content of this report.

## ACKNOWLEDGMENT

*The authors would like to thank Sir Andrew Haines for his comments and advice.*
